# Study on Rheological Properties and Pouring Process of Hydroxyl-Terminated Polybutadiene (HTPB) Propellants

**DOI:** 10.3390/polym15244707

**Published:** 2023-12-14

**Authors:** Haoyu Wang, Yongchao Ji, Xiaorui Jiang, Zhuo Li

**Affiliations:** 1 College of Science, Inner Mongolia University of Technology, Hohhot 010000, China; 20211100088@imut.edu.cn (H.W.); 20201000003@imut.edu.cn (Y.J.); 2 School of Civil Engineering, Hebei University of Engineering, Handan 056000, China

**Keywords:** viscoelastic fluid, propellant, rheological experiment, CFD simulation

## Abstract

The process of solid propellant production, which is the most widely used high-energy material, has garnered significant attention from researchers. However, there have been relatively few studies on its processing, due to the unique nature of the casting process. This paper aims to further analyze the pouring process of the propellant slurry. Initially, we obtained a sample of the propellant slurry and measured its rheological parameters using a rotary rheometer. From the analysis of the experimental results, we derived the viscosity parameters and the yield values of the propellant slurry. Subsequently, we simulated the pouring process, setting the slurry parameters based on the data obtained from the rheological measurement experiment. The simulation results demonstrated that the flower plate significantly impacts upon the cutting and separating effect on the slurry during pouring. Upon leaving the flower plate, the slurry descends onto the core mold platform under the influence of gravity, gradually flowing along the edge of the core mold. Although there may be some small voids in the pouring process, the voids will disappear completely at the end of pouring. A comparison with the actual pouring situation revealed a higher consistency between the simulation results and reality, thus establishing the reliability of the simulation method as a reference for analyzing the pouring process.

## 1. Introduction

In recent years, the growing demand for civil and military applications has led researchers to increasingly focus on solid high-energy materials. However, due to the potential adverse effects of energetic materials on the environment and health, thorough preparation has been necessary for their study. Moreover, the sensitive nature of these materials requires a meticulous research process [1]. Pouring is a crucial step in the production process of energetic materials [2]. Based on the aforementioned information, simulating the propellant pouring process has proven to be a highly cost-effective and convenient research approach. Composite solid propellants, typically utilized in solid rocket motors, consist of various solid particles and binders. The composition typically includes ammonium perchlorate (AP), hydroxyl-terminated polybutadiene (HTPB), aluminum, and other components such as plasticizers and curing agents [3,4,5,6,7,8,9,10]. These propellants exhibit high-energy contents, excellent combustion characteristics, and favorable storage and processing performances [11,12].

As a dependable and efficient energy storage material, the solid rocket propellant plays a crucial role in solid rocket motors—one of their primary components [13,14,15,16,17,18,19,20,21,22,23]. Ensuring the integrity of the propellant grain is essential during the production process, particularly during the pouring phase. It is imperative that the propellant slurry effectively fills all areas within the engine cavity. Any presence of holes or bubbles could compromise the grain’s integrity, potentially resulting in abnormal engine operation [24,25]. Studying the flow field in the pouring process holds significant engineering implications. The rheological properties of propellant slurry are highly intricate [26,27,28]. Generally, it demonstrates the nonlinear behavior of Bingham fluids, meaning that it possesses a yield value. In instances where the applied shear stress is below the yield value, there is an absence of flow [27]. When the shear stress surpasses the yield value, flow transpires, accompanied by a nonlinear correlation between viscosity and shear rate. In the pouring process, significant alterations in viscosity, yield value, and other parameters are expected [29,30,31,32,33].

Since the turn of the last century, researchers have delved into the rheological properties and pouring processes of propellant slurries. Notable contributors to this field include Tang, Kiage K, Muthiah Rm, and Sakovich G V [29,34,35,36]. However, due to a limited understanding of constitutive behavior in viscoelastic fluids and the still-evolving simulation technology, their investigations have mainly focused on propellant viscoelastic fluids. Nevertheless, their studies have laid a foundation for further research endeavors.

Over time, there has been increasing depth in the research and simulation analysis of the rheological properties of viscoelastic fluids, leading to a clearer understanding of the characteristics of propellant slurry. Du, Hai, Patel Mahesh, and other researchers have investigated the constitutive models and practical applications of various types of viscoelastic fluids [37,38,39,40,41].

Yang, Kanwar Pal Singh, Evan Mitsoulis, Wan, D.P. Mishra, Jiang, Wang, and other researchers studied and analyzed the rheological properties of propellant slurry by changing the physical properties of components, as well as their environmental parameters and solid content [11,42,43,44,45,46]. Concurrently, different constitutive relations have been utilized to describe the behavior of propellant slurry. Yiolanda Damianou, building upon existing constitutive relations, conducted numerical simulations to analyze and investigate the flow characteristics of propellant slurry under pressure [47].

Martinez-Pastor J et al. primarily focused on describing the rheological properties of propellant slurry. They adjusted the environmental parameters to simulate the conditions in the production environment, thereby establishing a foundation for real-world production [2]. In the light of advancements in the production of propellant slurry component particles, there has been a reduction in their diameter to the nanometer scale. Consequently, Jalpa A et al. conducted research on the rheology of propellant slurry-containing nanoparticles [1]. As rheometers continue to advance, dynamic rheological measurements have become increasingly accessible. In their study, LADE R et al. analyzed the dynamic rheological behavior of the propellant by employing a dynamic rheometer, which allowed them to obtain dynamic parameters such as the complex modulus of propellant slurry [12]. Li Xin et al. utilized a dynamic rheometer to conduct the rheological testing and characterization of a novel propellant slurry [48].

There are two primary approaches to studying the flow field during the pouring process: experimental and simulation methods. Experimental research offers the benefits of being intuitive and reliable, but it also comes with high costs and difficulties in capturing the intricacies of the flow field. On the other hand, simulation methods allow for a detailed analysis of the flow field, enabling the prediction of potential issues and the optimization of pouring process parameters. This method was both effective and cost-efficient. However, the current research on the flow field in the pouring process is still in its early stages, due to the complex rheological properties of the slurry.

Compared to China, Western countries have been pioneers in solid propulsion technology, having developed and matured their expertise over a longer period of time. In particular, the United States and Russia (formerly the Soviet Union) have achieved notable advancements in solid propulsion. Their advanced technology has found extensive applications in short-range missiles, intercontinental missiles, and even civilian aerospace applications [25,40].

**Figure 1:**
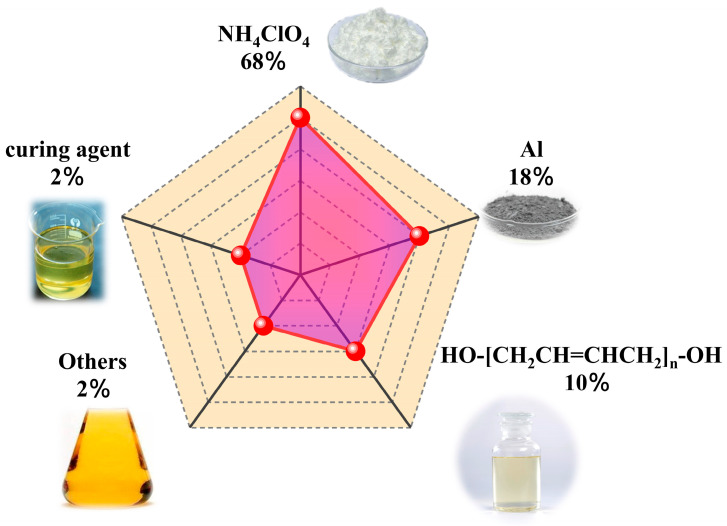
Schematic diagram of HTPB propellant slurry formulation.

In this study, the research focused on a HTPB propellant with a solid content of over 70% [49,50,51,52,53], as shown in Figure 1. Experimental measurements were conducted to determine the flow-dependent performance parameters of the propellant slurry. These parameters were then used to simulate the casting process of the propellant slurry slab and to analyze the flow field characteristics during casting.

## 2. Rheological Parameter Acquisition and Constitutive Construction

### 2.1. Rheological Measurement Experiment

According to whether or not they adhere to the Newtonian viscosity law, fluids are commonly classified as either Newtonian viscosity fluids or non-Newtonian viscosity fluids. Solid propellant slurry, a concentrated solution of polymer materials, belongs to the category of non-Newtonian fluids, which are typically influenced by external factors, in terms of their viscosity. Generally speaking, non-Newtonian fluids can be classified into four types based on the relationship between viscosity and shear rate: Newtonian fluids, expansive fluids (or dilatant fluids), pseudoplastic fluids, and Bingham fluids. Experimental measurements indicate that the propellant slurry exhibits the characteristics of a nonlinear Bingham fluid, specifically, a Bingham fluid with nonlinearity.

Measuring instruments such as viscometers and rheometers are commonly employed for the measurement of rheological parameters. Viscometers are considered to be excellent instruments due to their simplicity, durability, and easy operation. However, they have limitations in terms of the viscosity range that they can measure, being restricted to low shear rates. Consequently, viscometers are unable to accurately depict the true viscosity curve. In recent years, advancements in sensor accuracy and control systems have led to the emergence of new rheological measuring instruments, such as rotary rheometers, which have gained popularity among scholars. These rheometers offer a wider measurement range for temperature, rate, and strain, enabling a more precise determination of rheological parameters.

**Figure 2:**
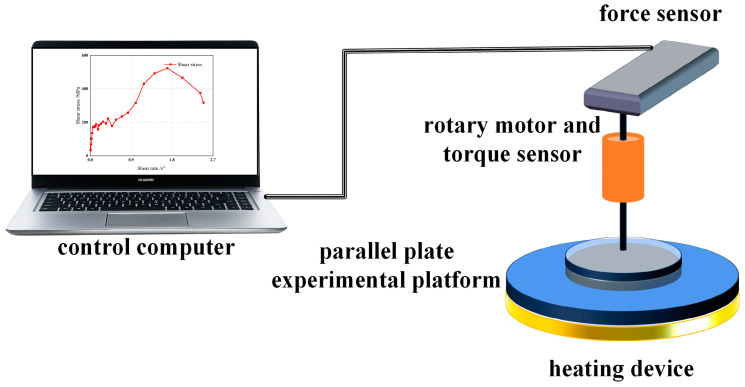
Schematic diagram of rotary rheometer.

**Figure 3:**
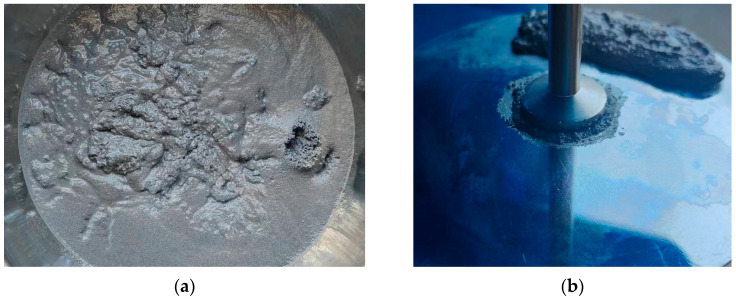
Physical image of the experimental process. (**a**) Drug slurry diagram; (**b**) Experimental diagram of a rotational rheometer.

The rotational rheometer is commonly employed to measure the viscosity of the propellant at various shear rates. Experiments are conducted using parallel plates, with the distance between the plates being controlled at 2 mm. The experimental platform is maintained at a temperature of 50 degrees Celsius. The shear rate is treated as an independent variable, ranging from 0.01 s^−1^ to 3 s^−1^. The original diagram of the experimental setup is illustrated in Figure 2. Figure 3 is the physical diagram in the rheological test.

### 2.2. Construction of a Rheological Constitutive Model

Previously, researchers have studied the rheological properties of other propellant slurries. Studies have shown that solid propellant slurry exhibits nonlinear Bingham fluid characteristics. The slurry has a yield value, and there is no flow when the applied shear stress is lower than the yield value [26,27,28].

Various viscosity models, including the power law model, Cross model, Carreau model, and Bingham model, are commonly utilized to describe the viscosities of viscoelastic fluids. In the Bingham model, when there is nonlinearity between the yield stress and shear rate, it is referred to as the Herschel-Bulkley model. The Herschel-Bulkley constitutive model characterizes fluid properties as follows: the fluid exhibits a yield value, and flow does not occur if the fluid shear stress is below this yield value. However, when the shear stress exceeds the yield value, flow is initiated and the fluid viscosity can be described by the power law model. Considering the properties of HTPB propellant slurry, it can be observed that the HTPB propellant behaves similarly to the Herschel-Bulkley model. Therefore, this model is more suitable for describing the rheological properties of HTPB propellant, as it offers a relatively simple mathematical form and fewer parameters, facilitating engineering applications. In addition, researchers have proposed several improved models based on the Herschel-Bulkley constitutive model to accommodate different viscoelastic fluids.

When calculating small and large deformations, viscosity divergence is a recurring issue. To accurately depict the nonlinear relationship between the shear stress and shear strain of viscoelastic fluids under large strain conditions, and to prevent the numerical divergence of the equivalent viscosity coefficient in the Bingham model and its derivative model, the Herschel-Bulkley-Papanastasiou (HBP) model is employed to characterize the rheological properties of propellant slurry. Proposed by Papanastasiou [21], the HBP model approximated the HB model using a continuous function. Subsequently, Frigaard et al. [22] confirmed that this model effectively solves the divergence problem encountered when using the equivalent viscosity coefficient for small deformations. The HBP model and its expression for the equivalent viscosity coefficient are as follows:

$$\eta =\eta_{0}{\left(\dot{\gamma }\right)}^{n-1}+\frac{\tau_{y}}{\dot{\gamma }}\left(1-e^{-m\dot{\gamma }}\right)$$

In the formula, *η*_0_ is the shear viscosity, *τ*_y_ is the yield stress, *γ* is the shear rate, and n and m are the control parameters.

**Figure 4:**
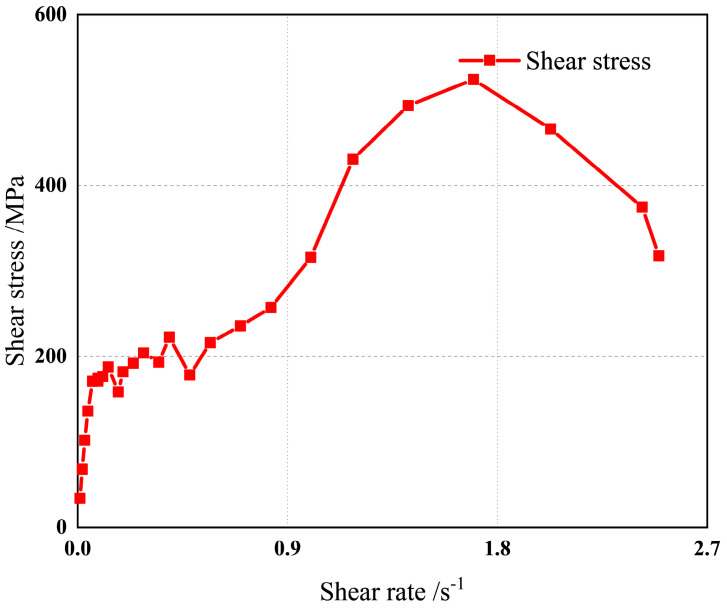
Experimental measurement diagram of yield value.

The point in the Figure 4 is the experimental data point. The shear stress corresponding to the first inflection point of the curve in the figure is the yield value of the propellant slurry, 180 MPa, as shown in Figure 4. A series of shear rates were selected to measure the viscosity of the slurry at a constant shear rate. The data obtained from the comprehensive experiment are fitted by the Herschel-Bulkley-Papanastasiou constitutive model, and the viscosity constitutive is obtained as follows. By observing the experimental data, it is demonstrated that the viscosity gradually decreases with the increase of the shear rate, and the viscosity changes greatly when the shear rate is small (that is, less than 0.2). When the shear rate is large (that is, greater than 0.2), the viscosity changes little and the viscosity is low—less than 1000 Pa · s.

In the formula, *η*_0_ is the shear viscosity, *τ*_y_ is the yield stress, γ is the shear rate, and n and m are the control parameters.

The fitted viscosity constitutive parameters are shown in Table 1, which are substituted into the above viscosity constitutive equation; that is, the propellant viscosity constitutive. The fitting accuracy is high: the R^2^ value is 0.978.

## 3. Pouring Simulation Analysis

### 3.1. Physical Model and Basic Assumptions

In the production process of a solid rocket motor, three methods are often used to improve the pouring quantity. Firstly, the vacuum extraction of the pouring environment is carried out to reduce the formation of bubbles. The second method is to design a reasonable flower plate that can make the propellant slurry flow down more finely and avoid the formation of gaps in the stacking process. Finally, the temperature is controlled to keep the rheological properties of the slurry stable, so as to stabilize the pouring process. These methods can effectively maintain the stability of the slurry and improve the pouring quantity. Therefore, in the simulation study, the environmental pressure is set to one-tenth of the standard atmospheric pressure, and no heat exchange is performed, to simulate the real environment as much as possible and to improve the accuracy of the simulation.

**Figure 5:**
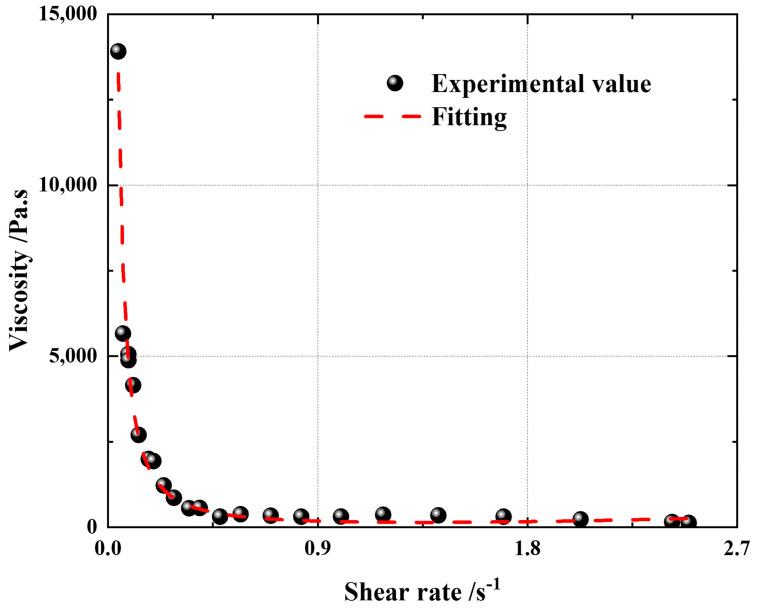
Variable shear rate viscosity diagram.

Commonly used propellant slurry pouring methods include the intubation pouring method, the vacuum pouring method, and the bottom pouring method. Among them, the vacuum casting method is commonly used in the production of solid rockets. In this study, the vacuum pouring method was used to pour the propellant. This pouring system is shown in Figure 5. The pouring system mainly includes a hopper, hose valve, pouring cylinder, combustion chamber, shell, core mold, flower plate, automatic weighing device, and other supporting facilities.

During the pouring process, the slurry is first injected into the hopper, and the hose valve is closed. Then, the air pressure in the pouring cylinder is reduced to about 900 Pa via vacuum pumping, and the atmospheric pressure at the location of the experiment is maintained above the hopper, at about 87,400 Pa.

**Figure 6:**
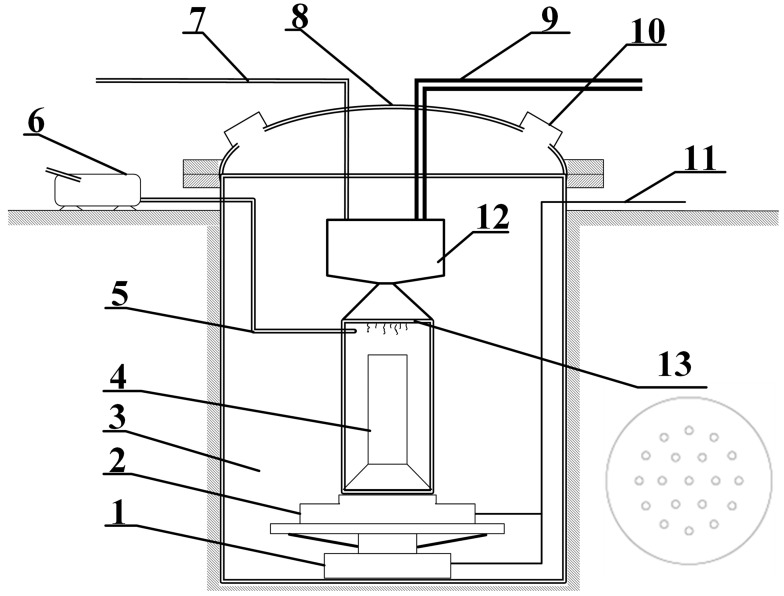
Schematic diagram of gating system. 1. Lifting device. 2. Weighing device. 3. Casting cylinder. 4. Core mold. 5. Vacuum pump pipeline. 6. Flower plate. 7. Vacuum pump. 8. Pressure pipeline. 9. Casting cylinder head. 10. Feed pipe. 11. Observation window. 12. Control circuit. 13. Hopper.

When the hose valve is opened, the slurry flows into the lower engine shell under the action of pressure difference and gravity. When the pulp passes through the flower plate, it is divided into thin strips, which will increase the surface area and facilitate degassing. At the same time, this also makes the slurry flow more evenly into the entire cross-sectional area of the shell, reducing local accumulation, which is conducive to leveling. The pouring device is shown in Figure 6.

In this study, the external diameter of the engine is 108 mm, and the final height of the grain is 136 mm. When the slurry is poured, the slurry flows out of the pipe and falls into the cavity after being cut and divided by the flow plate. The plate is a porous structure, the holes are circular, the diameter of the holes is 5 mm, the thickness of the plate is 5 mm, the number of holes is 19, and it is evenly distributed in the ring. The core mold is a cylinder, and the bottom is gradually enlarged; this is located in the center of the engine. The model is a cylinder, which is a symmetrical structure.

### 3.2. Boundary Conditions

Because the pouring space and the flower plate are symmetrical structures, and in order to reduce the calculation time and the number of calculations, the quarter of the model is taken for calculation, and the section is set to the symmetrical boundary. The bottom and the outside of the model are non-shear wall boundaries.

Due to the small size of the engine, the pouring time is expected to be 15–20 min. Now, 18 min is selected as the total time of simulation pouring. For the boundary of the pouring inlet, it is set as the mass flow rate inlet, and the final poured amount and pouring time are determined to be 0.316 g/s and 1080 s, respectively.

### 3.3. Computational Solution Method

#### 3.3.1. Finite Volume Method

The simulation process is carried out via the finite volume method. In general, there are many methods for solving partial differential equations in fluid problems, including the finite difference method (FDM), the finite element method (FEM), the spectral method, and the meshless method. The main purpose of these methods is to approximate the analytical solution of continuous partial differential equations using discrete equations. In addition to the meshless method using the point cloud for spatial discretization, other methods need to construct a spatial grid system connected by nodes. The refinement of the grid represents that the discrete solution gradually approaches the exact solution.

As a numerical calculation method, the finite volume method has the advantages of high calculation accuracy, a wide application range, and a high calculation efficiency. It uses the average value in the control volume to describe the physical quantity, avoids the interpolation error in the finite element method, and is suitable for unstructured grids, which are more suitable for the more complex free surface problem of slurry pouring.

#### 3.3.2. Liquid Level Tracking Method

The simulation process uses the liquid level tracking method (VOF). In this method, the incompatible fluid components share a set of momentum equations, and the phase volume fraction F is introduced to track the interphase interface in the computational domain, where F denotes the ratio of the volume of one phase to the volume of the grid.

The model simulates two or three immiscible fluids by solving a separate momentum equation and dealing with the volume ratio of each fluid passing through the region. Typical applications include fluid jetting, bubble motion in the fluid, fluid flow at the dam mouth, steady-state, and the transient treatment of the gas–liquid interface. Generally speaking, VOF is mainly applicable to unsteady multiphase flow models, and it can only be applied to the steady-state problems of multiphase flow models for certain specific problems.

The VOF method can construct the interface by calculating the phase fraction of each grid element in the whole computational domain.

The mass continuity equation and momentum equation are represented by the following two formulas.

$$F_{DIF}+F_{SOR}=\frac{\partial F}{\partial t}+\frac{1}{V_{F}}[\frac{\partial }{\partial x}\left(FA_{x}u\right)+R\frac{\partial }{\partial y}\left(FA_{y}v\right)+\frac{\partial }{\partial z}\left(FA_{z}w\right)+\xi \frac{FA_{x}u}{x}]$$

$$\begin{array}{l} \frac{\partial u}{\partial t}+\frac{1}{V_{F}}\left\{uA_{x}\frac{\partial u}{\partial x}+vA_{y}R\frac{\partial u}{\partial y}+wA_{z}\frac{\partial u}{\partial z}\right\}-\xi \frac{A_{y}v^{2}}{xV_{F}}=-\frac{1}{\rho }\frac{\partial P}{\partial x}+G_{x}+f_{x}-b_{x}\\ \;\;\;\;\;\;\;\;\;\;\;\;\;\;\;\;\;\;\;\;-\frac{R_{SOR}}{\rho V_{F}}\left(u-u_{w}-\delta u_{s}\right)\\ \frac{\partial v}{\partial t}+\frac{1}{V_{F}}\left\{uA_{x}\frac{\partial v}{\partial x}+vA_{y}R\frac{\partial v}{\partial y}+wA_{z}\frac{\partial v}{\partial z}\right\}+\xi \frac{A_{y}uv}{xV_{F}}=-\frac{1}{\rho }\left(R\frac{\partial P}{\partial y}\right)+G_{y}+f_{y}-b_{y}\\ \;\;\;\;\;\;\;\;\;\;\;\;\;\;\;\;\;\;\;\;-\frac{R_{SOR}}{\rho V_{F}}\left(v-v_{w}-\delta v_{s}\right)\\ \frac{\partial w}{\partial t}+\frac{1}{V_{F}}\left\{uA_{x}\frac{\partial w}{\partial x}+vA_{y}R\frac{\partial w}{\partial y}+wA_{z}\frac{\partial w}{\partial z}\right\}=-\frac{1}{\rho }\frac{\partial P}{\partial z}+G_{z}+f_{z}-b_{z}\\ \;\;\;\;\;\;\;\;\;\;\;\;\;\;\;\;-\frac{R_{SOR}}{\rho V_{F}}\left(w-w_{w}-\delta w_{s}\right) \end{array}$$

The left two terms of the mass continuity equation equality are:

$$F_{DIF}=\frac{1}{V_{F}}\{\frac{\partial }{\partial x}\left(v_{F}A_{x}\frac{\partial F}{\partial x}\right)+R\frac{\partial }{\partial y}\left(v_{F}A_{y}R\frac{\partial F}{\partial y}\right)+\frac{\partial }{\partial z}\left(v_{F}A_{z}\frac{\partial F}{\partial z}\right)+\xi \frac{v_{F}A_{x}F}{x}\}$$

$$\frac{F_{SOR}}{F}=\frac{\partial }{\partial x}\left(uA_{x}\right)+R\frac{\partial }{\partial y}\left(vA_{y}\right)+\frac{\partial }{\partial z}\left(wA_{z}\right)+\xi \frac{uA_{x}}{x}$$

The VOF equation used is:

$$F_{DIF}+F_{SOR}=\frac{\partial F}{\partial t}+\frac{\left[\frac{\partial }{\partial y}\left(FA_{x}u\right)+R\frac{\partial }{\partial y}\left(FA_{y}v\right)+\frac{\partial }{\partial z}\left(FA_{z}w\right)+\xi \frac{FA_{x}u}{x}\right]}{V_{F}}$$

$$F_{DIF}=\frac{1}{V_{F}}\{\frac{\partial }{\partial x}\left(v_{F}A_{x}\frac{\partial F}{\partial x}\right)+R\frac{\partial }{\partial x}\left(v_{F}A_{y}R\frac{\partial F}{\partial y}\right)+\frac{\partial }{\partial z}\left(v_{F}A_{z}\frac{\partial F}{\partial z}\right)+\xi \frac{v_{F}A_{x}F}{x}\}$$

## 4. Simulation Results Analysis

In the process of the pouring simulation, the slurry flows into the pouring space according to the constant mass flow rate. From an overall evaluation, during the whole pouring process, the total quantity of pouring increased steadily, but the quality growth rate slowed down slightly. On the one hand, due to the curing reaction during the pouring process, the viscosity of the slurry increased slightly, and the flow rate of the slurry decreased. On the other hand, in the pouring experiment, there will be a certain degree of accumulation near the plate hole or valve due to the viscosity of the slurry, resulting in a slow flow. Due to the above two reasons, the mass flow rate of the slurry is continuously reduced, and the mass increment of the slurry in the pouring space is continuously reduced. Through the final results, it can be seen that the amount of poured slurry in the experiments was not significantly different from the amount in the simulation.

Because the calculation method in the simulation was the finite volume method, some small liquid regions were ignored. Thus, the variation rate of the amount of poured slurry in the simulation deviated from the preset amount, but the deviation was small. The final pouring quantity of the experiment was 1.39029 kg, which was greater than the 1.37805 kg of the simulation analysis, that is, the experiment poured 0.01224 kg more slurry, and the simulation error was 0.88%. The volume of the pouring area in the simulation model is equal to the volume of the actual pouring engine test. In theory, the total mass of the pouring slurry should be equal, but after the experiment, it is found that there is residual slurry at the valve, the nozzle or other positions, resulting in the actual pouring slurry being slightly more than the simulation value. Because of the existence of propellant slurry residue, in order to maintain the quantity of the final casting product, in line with the design, the pouring time will be extended. The final product quantity meets the requirements.

**Figure 7:**
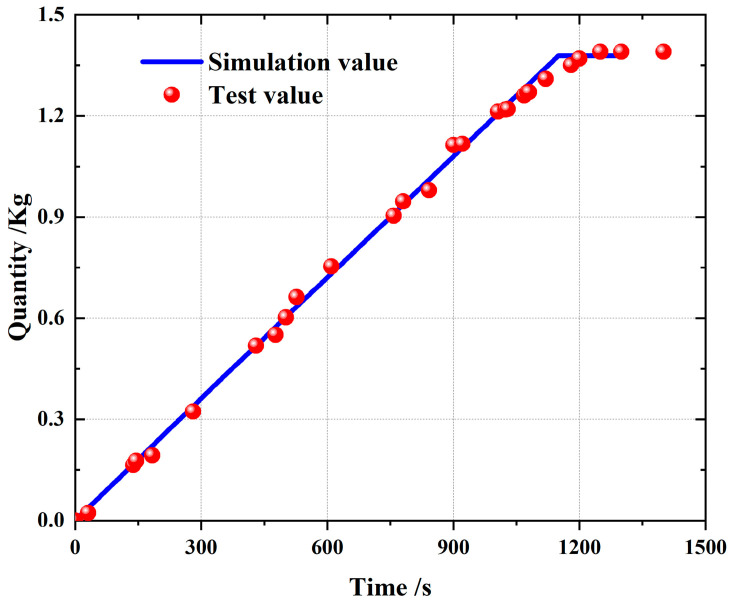
Slurry casting quantity comparison diagram.

Observing the quantity comparison of the slurry in Figure 7, 1080 s is the planned stop pouring time, but the actual test value is less than the simulation value at 1080 s because of the loss. As the experiment is still pouring, the final experimental measurement quantity is greater than the simulation quantity. These reasons described above lead to the quantity of the experimental pouring slurry being slightly higher than the simulation quantity. Figure 7 lists the relevant data comparison. From a comparison between the time of the test and that of the simulation for completing the pouring, it is found that the time of the simulation to complete the pouring is slightly shorter than the time of the experiment to complete the pouring, and that the error of the simulation pouring time relative to the experimental pouring time is 8.08%.

**Figure 8:**
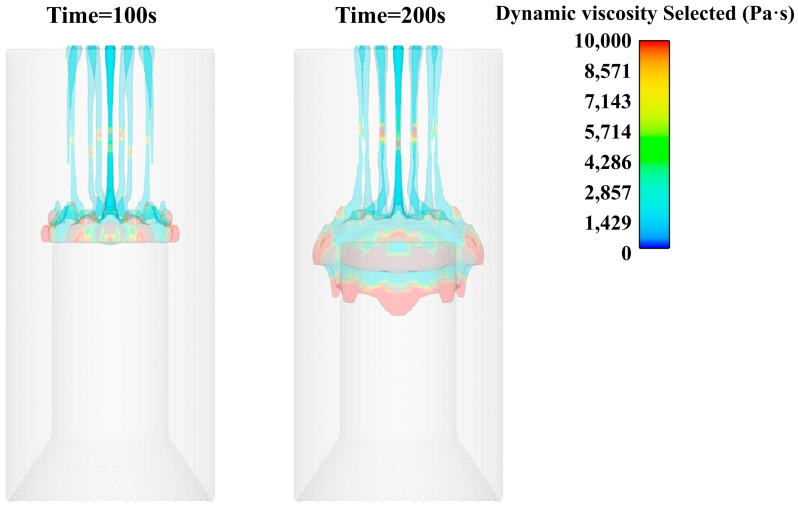

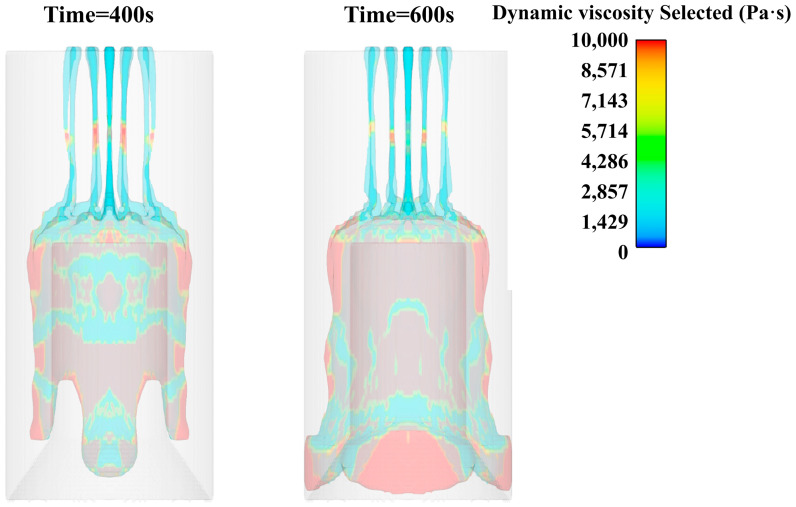
Viscosity cloud diagram of slurry pouring (100 s, 200 s, 400 s, 600 s).

Through the simulation results, it can be found that the cutting of the slurry through the flower plate hole increases the shear rate of the slurry through the flower plate, thereby reducing the viscosity of the slurry and making it pass through the flower plate more smoothly, as shown in Figure 8.

The slurry falls onto the central platform under the action of gravity after passing through the flower plate. At the same time, due to the action of gravity, the slurry is elongated in the middle position, and the shrinkage phenomenon occurs. However, because the lower slurry has been in contact with the core mold platform, the continuous elongation of the slurry is prevented, so that there is no large area of the slurry fracture phenomenon, as shown in Figure 8.

**Figure 9:**
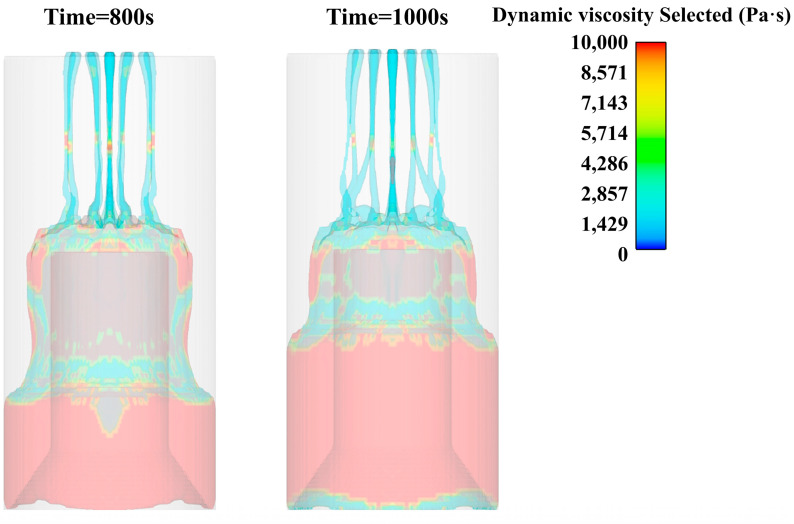

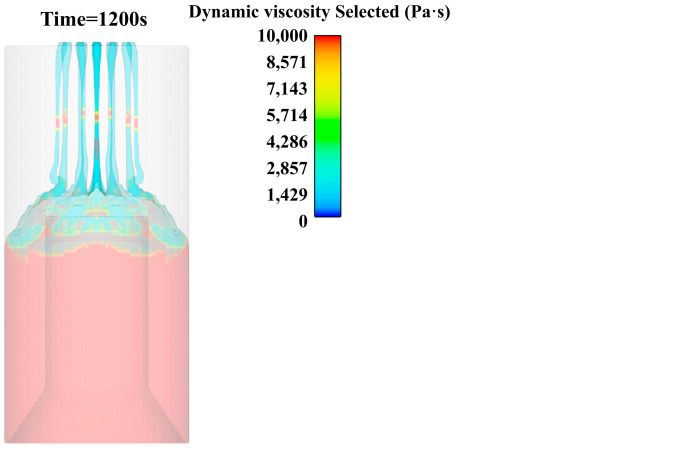
Viscosity cloud diagram of slurry pouring (800 s, 1000 s, 1200 s).

When the slurry accumulates to a certain amount on the core mold platform, the edge is gradually smooth under the action of surface tension, and the thickness of the edge slurry is increasing. When the surface tension of the edge slurry is not enough to support it, the slurry gradually slips along the edge of the platform and drives the slurry to slip at other locations, as shown in Figure 9.

**Figure 10:**
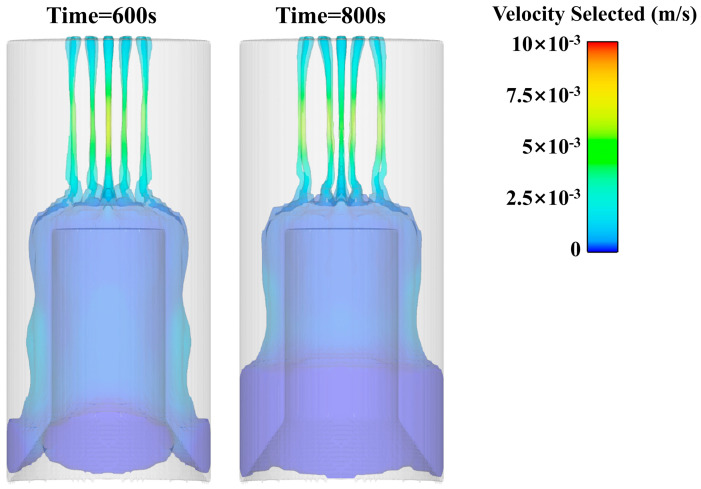
600 s and 800 s Velocity cloud diagram of slurry pouring.

After analyzing the flow pattern of the slurry, the flow properties of the slurry are analyzed. Usually, the lower limit of the Reynolds coefficient is relatively stable, and it is used to determine the flow pattern. Here, we choose 800 s of fluid data for calculation, as shown in Figure 10.

The lower limit calculation formula of the Reynolds coefficient:

Re=ρVR/μ

In the formula, Re is the lower limit of the Reynolds coefficient; *ρ* is the fluid density, 1800 kg/m^3^; *V* is the fluid velocity, 0.005 m/s; *R* equals 0.108 m, which is the characteristic length (maximum diameter of the pouring cylinder); and *μ* equals 1500 kg/m∙s, which is the viscosity.

The lower limit of the Reynolds number is 0.000648, which is less than the critical Reynolds number. Therefore, we judge that the pouring process is laminar flow.

**Figure 11:**
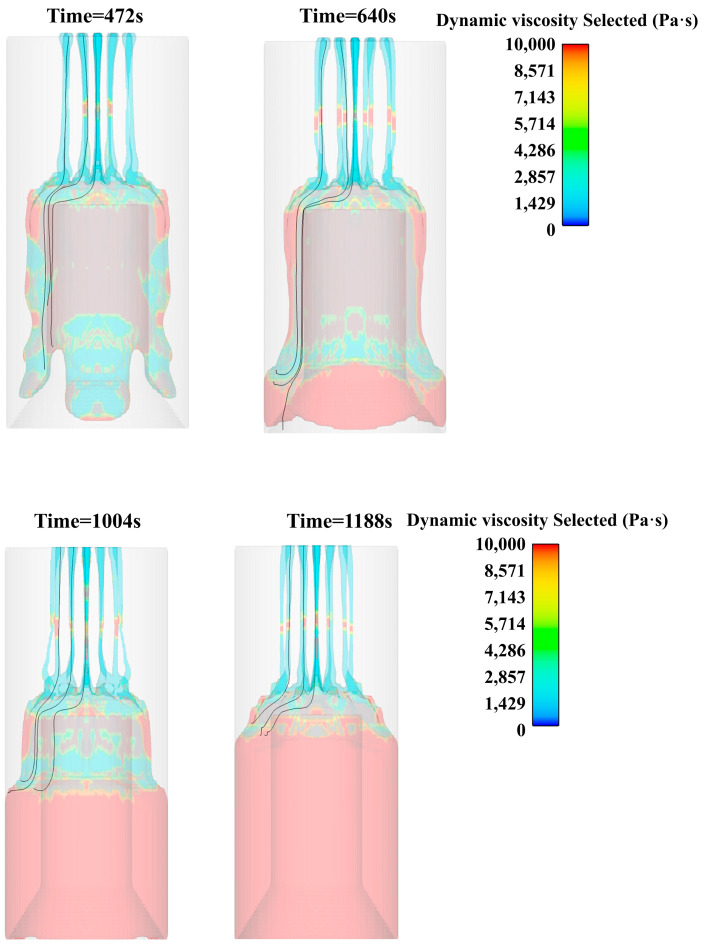
Slurry flow chart at different times.

Through the observation of the viscosity distribution cloud map during the pouring process, it can be found that the slurry flow is basically laminar flow. The post-processing software is used to present the slurry flow line of the three layers of the flower plate, and the slurry flow is characterized in more detail. From an observation of the change law of the propellant streamline during the pouring process, it was found that the slurry flowing out farther from the center of the plate accumulated closer to the edge of the core mold. At the same time, when the slurry flows downward along the edge of the core mold under the action of gravity, the slurry of the outer ring will also flow at the outermost edge, and the interior will flow at the innermost layer, showing laminar flow, as shown in Figure 11.

## 5. Conclusions

(1) Slurry casting simulations were performed, and the results were compared with the measured results. The total mass error between the simulations and experiments was 0.88%, and the casting time error was 8.08%. It was found that the simulation could describe the actual process accurately. Thus, simulations are effective for studying and improving the casting process.
(2) During the pouring process, the surface of the slurry accumulation is uneven. During the flow process, the phenomenon of the convergence of multiple fine strips and intermittent stretching-diameter shrinkage-fracture occurs locally. Under the action of gravity, the slurry gradually became compact and level, and no holes or bubbles appeared.
(3) In the pouring process, the slurry is basically laminar flow. The slurry piled up on the core mold will appear as obvious stratified flow in the process of downward flow.

## Figures and Tables

**Table 1:** Fitting viscosity constitutive parameters.

Parameter	*η*_0_	n	*τ*_y_	m
	133.982	−0.467	170.880	−0.169

## Data Availability

The data presented in this study are available on request from the corresponding author.
